# Sperm cryodamage occurs after rapid freezing phase: flow cytometry approach and antioxidant enzymes activity at different stages of cryopreservation

**DOI:** 10.1186/s40104-016-0076-x

**Published:** 2016-03-05

**Authors:** L. S. Castro, T. R. S. Hamilton, C. M. Mendes, M. Nichi, V. H. Barnabe, J. A. Visintin, M. E. O. A. Assumpção

**Affiliations:** Laboratory of Spermatozoa Biology, Department of Animal Reproduction, School of Veterinary Medicine and Animal Science, University of Sao Paulo, Sao Paulo, Brazil; Laboratory of In Vitro Fertilization, Cloning and Animal Transgenesis, Department of Animal Reproduction, School of Veterinary Medicine and Animal Science, University of Sao Paulo, Sao Paulo, Brazil; Laboratory of Andrology. Department of Animal Reproduction, School of Veterinary Medicine and Animal Science, University of Sao Paulo, Sao Paulo, Brazil

**Keywords:** Bovine, DNA integrity, JC-1, Sperm viability

## Abstract

**Background:**

In order to improve the efficiency of bovine sperm cryopreservation process, it is important to understand how spermatozoa respond to differences in temperature as well as the ability to recover its own metabolism. The combination between flow cytometry approach and antioxidant enzymes activity allows a more sensible evaluation of sperm cell during cryopreservation. The aim of this study was to evaluate sperm attributes and antioxidant enzymes activity during different stages of cryopreservation process. Semen samples from Holstein bulls (*n* = 4) were separated in 3 treatments: fresh (37 °C); cooled (5 °C); and thawed. Evaluation occurred at 0 h and 2 h after incubation. Membrane integrity, mitochondrial membrane potential (MMP) and DNA damages were evaluated by flow cytometry; activities of antioxidant enzymes such as catalase, superoxide dismutase and gluthatione peroxidase were measured by spectrofotometry.

**Results:**

There was an increase in the percentage of sperm with DNA damage in the thawed group, compared to fresh and cooled, and for 2 hs of incubation when compared to 0 h. Considering MMP, there was an increase in the percentage of cells with medium potential in thawed group when compared to fresh and cooled groups. Opposingly, a decrease was observed in the thawed group considering high mitochondrial potential. Also in the thawed group, there was an increase on cells with damaged acrosome and membrane when compared to fresh and cooled groups. Significant correlations were found between antioxidant enzymes activity and membrane or mitochondrial parameters.

**Conclusion:**

Based on our results, we conclude that cryopreservation affects cellular and DNA integrity and that the critical moment is when sperm cells are exposed to freezing temperature. Also, our study indicates that intracellular antioxidant machinery (SOD and GPX enzymes) is not enough to control cryodamage.

## Background

Sperm cryopreservation is an essential biotechnology for assisted reproduction, allowing the maintenance of male gametes for an undefined period. It helps to simplify the transport of genetic material and improve the logistic of breeding programs. When associated to other techniques, such as fixed time superovulation and in vitro embryo production, this technique enables fast genetic gain in the herd. Despite the large variability of extenders and cryoprotectants, the efficiency of cryopreservation is still limited to 40 to 50 % of cellular survival [1].

Reduced fertility of cryopreserved semen can be associated to changes in plasma membrane integrity and structure [2]. Giraud et al. [3] demonstrated that sperm membrane fluidity decreased during cryopreservation and the response of spermatozoa to a freezing protocol could be predicted by the status of membrane of fresh semen. It is characterized mainly by lipids profiles. Consequently, susceptibility to changes in cold temperature seems to be related to the ratio of polyunsaturated fatty acids (PUFA) [4].

A negative aspect of high amounts of PUFA in the sperm plasma membrane is the increased susceptibility to oxidative stress, since PUFA is easily oxidized. The reduced cytoplasm contributes in this scenario, with limited cytoplasmic content of antioxidant enzymes, such as superoxide dismutase (SOD) [5], and glutathione peroxidase (GPX) [6], that work together to protect the cell against free radicals normally formed by mitochondrial metabolism. Because of that, the main source of antioxidant for sperm is the seminal plasma [7]. However, in cryopreserved semen, this antioxidant machinery is extremely diluted, impairing the protective role. Then, we can assume that cryopreserved sperm are highly dependent of intracellular antioxidant machinery and understanding the behavior of this intracellular protection during the process of cryopreservation is important to improve this biotechnology.

The bioenergetics function of sperm mitochondria plays a significant role on reactive oxygen species (ROS) formation, which in sperm is especially important to capacitation, hyperactivation and acrosome reaction [8]. However, when an imbalance occurs between higher production of ROS and a decrease on antioxidant mechanisms, oxidative stress follows, with deleterious effects to sperm motility and fertility [9], due to membrane, acrosome and DNA damages [10]. This chromatin alteration normally is not related to the ability of sperm to penetrate the oocyte, but can directly affect embryonic development [11, 12].

Routinely, semen evaluation prior to the cryopreservation process in commercial set-ups only assesses motility, sperm morphology and concentration. However, this evaluation is not able to detect functional changes in plasma membrane, acrosome, mitochondria or DNA. These characteristics are essential to enable the subsequent fertilization of the oocyte and further embryonic development [13–15]. The use of fluorescent probes associated to the low complexity of flow cytometry allows a high throughput analysis of sperm characteristics, providing a more refined evaluation when compared to conventional analysis. In addition, the study of each compartment of the sperm cell combined with intracellular antioxidant enzymes allow us to understand the function of SOD, GPX and catalase, and how they respond during cryopreservation. Our hypothesis is that during this process, only membranes, mitochondrial potential and DNA are affected by temperature decrease, without alteration in antioxidant enzymes activity. Then, the aim of this study is to evaluate different sperm attributes such as chromatin damage, plasma and acrosome membrane integrity, mitochondrial membrane potential and intracellular antioxidant enzymes activity during different stages of cryopreservation process (fresh, cooled and thawed) in order to understand how this particular cell responds to the differences of temperature, and the further ability to recover its metabolism after cryopreservation at different incubation periods (0 and 2 hs).

## Methods

### Experimental design

**Fig. 1:**
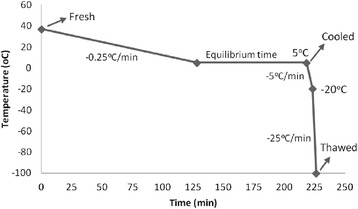
Graph displaying freezing curve with arrows indicating the point for evaluation of each sample group

Four adult (2 yr old) Holstein bulls were used, during 2 mon, for weekly sperm collections, using an artificial vagina (seven collections/bull). Animals were housed at the Department of Animal Reproduction from the School of Veterinary Medicine and Animal Science, University of Sao Paulo (VRA/FMVZ/USP). All procedures were performed according to the Bioethics Committee of the previously mentioned institution (protocol number 2094/2010). After immediate analysis (individual and mass motility and concentration), semen samples were diluted with commercial egg yolk extender Botubov® (Botupharma Ltda., Botucatu, SP, Brazil) to a final concentration of 20 × 10^6^ viable spermatozoa/mL. The same ejaculate was subjected to 3 treatments: fresh – evaluated immediately after extender addition at 37 °C; cooled – evaluated after equilibrium time, (5 °C/90 min); and thawed – evaluated after thawing. In the cooled and thawed groups, ten straws (0.25 mL) were loaded manually, and the cooling and freezing process were performed using a cryopreservation machine Cryogen® (Neovet, Uberaba, MG, Brazil) using the freezing curve described in Fig. 1. Before evaluations of the cooled group, each straw was incubated at 37 °C during 30 s. Considering the thawed group, straws were kept in liquid nitrogen, during 4 wk and thawed at 37 °C for 30 s. In each treatment, evaluations were performed at 0 h and 2 h after an incubation at 37 °C in water bath in Sp-TALP (Sperm Culture Tyrodes medium) [16].

### Antioxidant enzymes extraction

For the enzymatic antioxidant extraction, we used the protocol described by Hamilton et al. [17]. The extender was previously removed with a saline solution (0.9 % of NaCl) containing sucrose (7.5 %) and glucose (0.18 %). Briefly, 750 μL of semen were added to 3 mL of Sp-TALP and 1 mL of this solution was subsequently added to 7.5 mL of sucrose solution. Two centrifugations were performed (200G/5 min and 900G/10 min) and the supernatant was discarded. After the second centrifugation, the remaining 1 mL of solution was treated with 200 μL of Triton (4 %). After 30 min of incubation in a shaking water bath at 25 °C, samples were once again centrifuged (600G/8 mins) and the supernatant was removed and stored at −20 °C for further analysis.

### Enzymatic antioxidant activity

The enzymatic antioxidant activity of catalase, superoxide dismutase (SOD) and glutathione peroxidase (GPX) were quantified as previously described by Nichi et al. [18]. Briefly, catalase activity was assessed through measurement of hydrogen peroxide consumption. The reaction occurred at pH 8, 30 °C during 8 min. Enzymatic activity was measured using a spectrophotometer (230 nm wavelength). Absorbance was measured every 5 s, and the curve of H_2_O_2_ consumption was compared to a blank. Calculations used 0.071 L/mol·cm as the extinction coefficient for hydrogen peroxide. SOD activity was measured indirectly, through reduction of cytochrome c by superoxide (O_2_^•-^), generated by xanthine/xanthine oxidase system. SOD present in the sample will compete with cytochrome c by converting superoxide in hydrogen peroxide, thereby slowing the rate of cytochrome c reduction. Absorbance variation was accompanied for 5 min in a spectrophotometer fitted with a temperature regulator maintained at 25 °C (absorbance was measured every 5 s). GPX enzymatic activity was based on the consumption of NADPH; the reaction between hydrogen peroxide and reduced glutathione (GSH) that is catalyzed by the GPX together with the enzyme glutathione reductase (GR), is induced. This reaction causes the conversion of glutathione disulfide (GSSH – glutathione oxidized) to GSH, which in turn consumes NADPH. The consumption of NADPH was detected at a wavelength of 340 nm, for 10 min at 37 °C (measurements performed every 5 s). The results of GPX were expressed as units of GPX/ml of sample, and calculations used 6.22 L/mol·cm as the extinction coefficient of NADPH.

### Assessment of sperm DNA damage

Chromatin structure stability was analyzed based on *sperm chromatin structure assay* test (SCSA) [19], as described by Simões et al. [20]. This assay is based on an acid challenge that denatures DNA molecules from a susceptible chromatin structure, breaking hydrogen bounds and separating DNA strands, allowing acridine orange (AO) probe to intercalate and emit red (denatured single-stand break) or green (double stand DNA) fluorescent. The procedure was performed with 200,000 cells. Samples were incubated with TNE buffer (Tris–HCl 0.01 mol/L, NaCl 0.15 mol/L, EDTA 1 mmol/L and distilled water, pH 7.4) and acid detergent (HCl 0.08 mol/L, NaCl 0.15 mol/L, Triton X-100 0.1 % in distilled water, pH 1.2). After 30 s, AO solution were added (citric acid 0.1 mol/L, Na_2_HPO_4_ 0.2 mol/L, EDTA 0.001 mol/L, NaCl 0.15 mol/L, AO stock 6 μg/mL in distilled water pH 6), and each sample was analyzed by flow cytometry after 5 min of incubation at 37 °C, excited at 488 nm and detected at 630–650 nm (red) and 515–530 nm (green).

### Mitochondrial membrane potential

Mitochondrial membrane potential was evaluated by JC-1 probe (5,5',6,6'-tetrachloro-1,1',3,3' -tetraethyl- benzimidazolylcarbocyanine chloride, (Invitrogen, Eugene, OR, USA). This probe emits green fluorescent at low (LMP) and medium (MMP) mitochondrial potential or red-orange fluorescent at high potential (HMP). The procedure was performed with 200,000 cells diluted in SP-Talp and stained with JC-1 (76.5 μmol/L in DMSO). Samples were analyzed by flow cytometry after 10 min, excited at 488 nm and detected at 590 nm.

### Plasma membrane and acrosome integrity

Plasma membrane and acrosome integrity were evaluated by propidium iodide (PI) and fluorescein isothiocyanate-conjugated Pisum sativum agglutinin (FITC-PSA) respectively. This association divides sperm populations into four groups: intact membrane and intact acrosome (IMIA), intact membrane and damaged acrosome (IMDA), damaged membrane and intact acrosome (DMIA), damaged membrane and damaged acrosome (DMDA). The procedure was performed with 200,000 cells diluted in SP-Talp, stained with PI (0.5 mg/mL em NaCl 0.9 %) and FITC-PSA (FITC-PSA L-0770, Sigma, 100 μg/mL in sodium azide solution at 10 % in DPBS). Samples were analyzed by flow cytometry after 10 min, excited at 488 nm and detected at 630–650 nm (PI) and 515–530 nm (FITC).

### Flow cytometry analysis

Sperm samples analysis was performed by Guava EasyCyte™ Mini System (Guava® Technologies, Hayward, CA, U.S.A.) flow cytometry. This equipment contains a blue laser, which operates at 488 nm and emits a 20 mW visible laser radiation. A total of 10,000 events per sample were analyzed and data corresponding to yellow (PM1 photodetector – 583 nm), red (PM2 photodetector – 680 nm) and green fluorescent signals (PM3 photodetector – 525 nm) were recorded after a logarithmic amplification. For analysis, cell doublets and debris were excluded using PM3/FSC (forward scatter) and then dot plots used for DNA damage, JC-1 and FITC-PI assay were PM2/PM3, PM1/FSC and PM3/PM2, respectively. All data was analyzed by FlowJo® v8.7 software.

### Statistical analysis

Statistical analysis was performed using the software Statistical Analysis System 9.2 (SAS Institute, Cary, NC, USA). Data were tested for normality of residues and homogeneity of variances. Variables that did not comply with these statistical assumptions were subjected to transformations. Results were reported as untransformed means ± S.E.M. or median, minimum, maximum and quartiles (box plot) for parametric and non-parametric variables, respectively. High mitochondrial membrane potential was the only variable analyzed as non-parametric (WILCOXON PROC NPAR1WAY). PROC MIXED was used to evaluate the effect of treatment, incubation time and interaction between treatments x incubation time. Comparisons were performed using *least square means* (LS means). Person and Spearman correlations analysis were performed to verify the correlation between parametric and non-parametric variables, respectively (PROC CORR). All statistical analyses were calculated with a significance level of 5 %.

## Results

No interaction between treatment and incubation time was observed for any variables studied, indicating that groups behaved similarly for both 0 and 2 hs of incubation. Differences found on incubation periods were similar for all cryopreservation stages. Therefore, analysis were performed considering the effect of group and incubation periods separately.

### Enzymatic antioxidant activity

Considering enzymatic antioxidant activity of GPX and SOD, no difference was observed among groups (GPX: fresh = 178.27 (±22.10); cooled = 155.26 (±22.10); thawed = 150.81 (±22.44); *p* = 0.22. SOD: fresh = 167.42 (69.95; 181.98; cooled = 170.97 (65.50; 183.28); thawed = 166.65 (82.74; 183.44); *P* > 0.05). Catalase activity was not detected in any of the samples.

### Sperm DNA damage

**Fig. 2:**
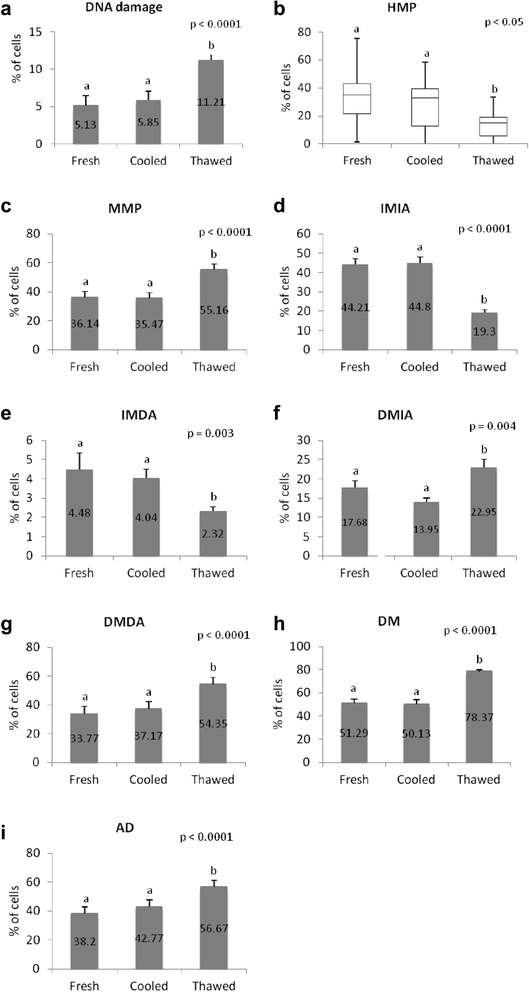
Evaluation of sperm profile separated by treatment group. **a** DNA damage (SCSAm); **b** High mitochondrial potential (HMP); **c** medium mitochondrial potential (MMP); **d** intact membrane and intact acrosome (IMIA); **e** intact membrane and damaged acrosome (IMDA); **f** damaged membrane and intact acrosome (DMIA); **g** damaged membrane and damaged acrosome (DMDA); **h** damaged membrane (DM); **i** damaged acrosome (DA). *ab* Different letters indicate differences between treatments

**Fig. 3:**
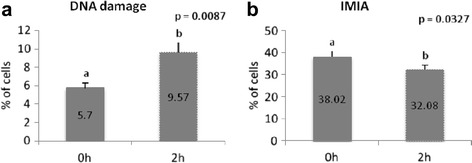
Evaluation of sperm profile separated by incubation period. **a** DNA damage (SCSAm); **b** intact membrane and intact acrosome (IMIA). *ab* Different letters indicate differences between incubation time

An increase in the population of sperm with DNA damage was found in the thawed group when compared other groups (Fig. 2a), and after 2 hs incubation when compared with cells in the initial (0 h) evaluation (Fig. 3a).

### Mitochondrial membrane potential

The percentage of spermatozoa with HMP was higher in fresh and cooled groups compared to thawed group (Fig. 2b). The opposite occurred in cells with MMP, which increased in the thawed group, when compared to the fresh and cooled groups (Fig. 2c). This data suggest that mitochondrial membrane potential was significantly impaired during the process of cryopreservation.

### Plasma membrane and acrosome integrity

Samples of thawed group had an increase in the percentage of cells with DMIA (Fig. 2f) and those with DMDA (Fig. 2g), when compared to both, fresh and cooled groups. These data are complementary to the decrease observed in the percentage of cells with intact membrane and acrosome in the thawed group, compared to the fresh and cooled groups (Fig. 2d). Membrane damage is more evident when compared to acrosome damage, with an increase of 56 % versus 33 %. Considering the incubation time, there was a decrease in the percentage of cells with intact membrane and acrosome, between 0 and 2 hs, regardless of the group (Fig. 3b).

### Correlations between enzymatic activities and sperm attributes

Correlations between SOD and GPX, mitochondrial membrane potential (high, medium and low) and intact membrane and intact acrosome (IMIA) were analyzed for each group separately (fresh, cooled and thawed; Table 1). Only correlation rates with significance of *p* < 0.05 were included in the table. For all treatments, GPX activity correlated negatively with IMIA and positively with MMP, LMP correlated negatively with HMP and positively with MMP. In the fresh group, there was a positive correlation between SOD activity and HMP, and a negative correlation between IMIA and MMP. In the cooled group, negative correlations were observed between GPX activity and HMP, SOD activity and MMP, IMIA and MMP and HMP and MMP. IMIA correlated positively with SOD activity and HMP. In the thawed group, there was a negative correlation between SOD activity and IMIA and a positive correlation between HMP and IMIA.

**Table 1:** Correlation (*r*) and significance level (*p*) between measured variables

	Fresh					Cooled					Thawed				
	SOD	HMP	MMP	LMP	IMIA	SOD	HMP	MMP	LMP	IMIA	SOD	HMP	MMP	LMP	IMIA
GPX	ns	ns	0.448**	ns	−0.530**	ns	−0.422**	0.465**	ns	−0.527**	ns	ns	0.338*	ns	−0.425**
SOD		0.362*	ns	ns	ns		ns	−0.383*	ns	0.399*		ns	ns	ns	−0.424**
HMP			ns	−0.705**	ns			−0.394*	−0.388*	0.510**			ns	−0.464**	0.469**
MMP				−0.360*	−0.541**				−0.690**	−0.420**				−0.960**	ns
LMP					ns					ns					ns

*GPX* glutathione peroxidase, *SOD* superoxide dismutase, *HMP* high mitochondrial potential, *MMP* medium mitochondrial potential, *LMP* low mitochondrial potential, *IMIA* intact membrane and intact acrosome

*ns* not significant

*P < 0.05 and **P < 0.01

## Discussion

Cryopreservation process lead to damages caused mainly by the cold shock, intracellular ice crystal formation, oxidative stress, solution effect and reorganization of lipids and proteins from the membranes [4]. In our study, using a very sensible flow cytometry approach combined with intracellular antioxidant enzymes evaluation, data suggest that the most significant impact on sperm cell during cryopreservation is actually when spermatozoa is exposed to temperatures lower than 5 °C, probably during fast freezing period, compromising membranes (plasma, acrosomal and mitochondrial) and DNA integrity. This quality is not transitory and non-reversible, as it cannot be recovered after 2 hs of incubation. Interestingly, antioxidant enzymes cannot repair the damage caused by cryopreservation, since their activity is unchanged during the process. Probably they act as a buffer system only to maintain the intracellular homeostasis from physiological sperm metabolic processes.

### Cryopreservation effects on plasma membrane and acrosome integrity

The stress on plasma membrane during sperm cooling may occur due to changes in the asymmetry of the phospholipid bilayer and the altered functional state of the membrane. Lipids and proteins in a fluid state, solidifies into gel, producing a rigid and fragile structure, more sensitive to injuries [21]. Furthermore, sperm cells are exposed to a hyperosmotic environment that induces an influx of water and ions across the membrane, leading to cell dehydration [22]. Our study shows that a progressive loss of integrity occurred in both acrosome and plasma membranes during cryopreservation. Interestingly, no differences were found when we compare fresh sperm and those evaluated after the cooling rate (5 °C). Evaluations of cooled group were performed after equilibrium period (Fig. 1), when sperm cells had already been exposed to the toxic effect of the cryoprotectant. Sperm damage was observed only when cells were exposed to freezing temperatures, indicating that intracellular crystal formation is probably more deleterious to the spermatozoa than the solution effect and cell dehydration. This could justify why sperm from other domestic species do not tolerate well cryopreservation processes. Susceptibility to cold temperatures and differences among species concerning sperm survival seems to be linked to the ratio of unsaturated and saturated fatty acid content [23], specially related to the proportion of cholesterol in the plasma membrane [24].

Sperm membrane injuries were more evident in the plasma membrane, with a decrease of 56 % on the percentage of intact cells in contrast to the 33 % decrease observed for acrosome integrity after cryopreservation. Damaged acrosome in cryopreserved sperm could be related to an event known as cryocapacitation, when alterations on membrane fluidity externalize inner phospholipids [2, 25] inducing premature acrosome reaction. This capacitation-like changes would, in turn, reduce sperm lifespan [26].

### Cryopreservation effects on mitochondrial membrane potential

We observed a shift from sperm with high mitochondrial potential to sperm presenting medium potential in the thawed group when compared to fresh or cooled groups. Correlations between mitochondrial membrane potential populations indicate in all treatments that high and medium potential were negatively correlated to low potential. It was expected that high and medium potential had a positive correlation, however in the cooled group, a negative correlation was observed. This suggest that mitochondrial activity decreases during cryopreservation, and probably the critical shift of population from high to medium potential occurs during the cooling phase, when the sperm is not suffering yet from crystal ice formation, but already have a decrease of its metabolism. This would confirm the results found by other authors that affirm that this process may strongly impair mitochondrial function [27, 28].

Similarly, to the plasma membrane, mitochondrial membranes may also suffer injuries due to crystal ice formation, which could explain the negative correlation found between MMP and IMIA in fresh and cooled groups. Considering the similarities regarding lipid and protein composition between plasma and mitochondrial membranes, the latter would be prone to similar damages induced by the cryopreservation process [29–31]. However, serious consequences would arise in case of mitochondrial membrane damages. A leakage of ions and free radicals from the mitochondria would decrease the electric potential, exposing the intracellular space, membrane lipids and DNA to a pro-oxidative environment [32]. Similar results were found by Schober et al. [27] that evaluated the effect of cryopreservation on mitochondrial function of equine sperm. These authors verified a decline on mitochondrial membrane potential and an increase in the oxidation rates by cytochrome c^2+^ oxidase, suggesting a partial rupture of cellular and mitochondrial membranes. Furthermore, microscopic examination of thawed mouse spermatozoa showed an increased relative area of matrix and swelling of the membrane with loss of mitochondria crystae [33]. Such structural damage is a possible explanation to the motility loss, frequently observed after semen cryopreservation which is around 50 % even in the most efficient protocols [1, 34].

### Cryopreservation effects on DNA integrity

DNA damage evaluation showed an increase in the percentage of cells with chromatin structure alterations in the thawed group, even after 2 hs of incubation. These data suggest that sperm chromatin structure become more susceptible to denaturation after cryopreservation, independently of the process stage. Castro et al. [9] demonstrated that bovine sperm when exposed to an oxidative environment has DNA impair, with further impact on embryo development. The thawing process also induce oxidative damages due to the fast increase in oxygen consumption after resumption of the metabolism, which was interrupted by cryopreservation [35]. In human sperm, Thomson et al. [36] demonstrated that DNA damage caused by cryopreservation process is mediated by oxidative stress and not by apoptosis. Thus, in our study, despite the lack of evaluation of ROS production, we speculate that one of the causes for the increased susceptibility of DNA to fragmentation during cryopreservation process and after 2 hs of incubation could be ROS production. Further studies should be performed aiming to confirm the impact of ROS on sperm DNA during the cryopreservation process.

Some authors described a correlation between SCSA positive spermatozoa and fertility [37, 38], as low DNA fragmentation index had increased percentages of AI success. Nevertheless, in spite of the low percentage of cells that were positive for DNA damage in our study, this result was similar to other studies in bovine species [20, 39–41]. Based on that, we suggest that bovine chromatin packaging is more efficient than other species such as human, in which high levels of DNA fragmentation are frequently observed [42]. Slowinska et al. [43] already demonstrated that bovine sperm DNA is comparatively less susceptible to the cryopreservation injuries than other species, such as boar. A hypothesis that could explain such low susceptibility to DNA denaturation is the high genetic selection pressure based on reproductive index, which bulls showing poor semen quality and subfertility are discarded from the breeding programs population, indirectly selecting animals with low DNA fragmentation susceptibility.

### Cryopreservation effects on enzymatic antioxidant activity

Dramatic metabolic changes are known to occur in the sperm during freezing [44, 45]. Several studies reported an increase on ROS production and specially lipid peroxidation after cryopreservation in different species, also impairing cell viability, motility and mitochondrial membrane potential [46–48]. The main source of antioxidant protection to the spermatozoa is the seminal plasma [7]. Its antioxidant machinery compensates the deficiency of cytoplasm antioxidant enzymes, which occurs due to cytoplasm content lost that happens during spermatogenesis. In our study, no differences between treatment and incubation period for intracellular SOD and GPX activities were found after cryopreservation. Reports in other species, such as ram [49] and fowl [50] also demonstrated no difference between fresh and thawed semen for intracellular GPX. Also, no detectable activity of catalase could be observed in our samples, in agreement with others studies [18, 51]. Our results indicate that during cryopreservation, intracellular antioxidant machinery remains unchanged regardless of the injury suffered, which is expected for a mature sperm cell that is not able to synthesize new proteins. Probably, the function of intracellular antioxidant enzymes is only to support physiological metabolism and basal ROS production by sperm mitochondria, and their amounts inside the cell are not enough to hold major oxidative damages.

Although there is no difference between cryopreservation stages, interesting correlations were observed between antioxidants enzymes activities and sperm functional profile. The GPX activity was positively correlated with medium mitochondrial potential (MMP) and negatively correlated with the percentage of sperm showing intact membrane and acrosome (IMIA) in all steps of cryopreservation process, which support the idea that this enzymatic machinery is responsible for the control of the ROS formed during aerobic respiration. Nichi et al. [18] demonstrated that GPX activity was higher in *Bos taurus* when compared to *Bos indicus* bulls. The increased GPX activity could be related to higher level of ROS in *Bos taurus* bulls in response to the heat stress. Similarly to our study, GPX activity also correlated negatively with membrane integrity of avian cryopreserved sperm [52]. Based on these findings and our results, we suggest that intracellular sperm GPX could be a marker of sperm injury in stressing situations, such as cryopreservation and heat stress, associated with loss of membrane integrity and increased number of cells with impaired mitochondrial membrane potential. Then, we can assume that GPX intracellular protection is not enough to protect cryopreservation damage.

Correlations between SOD activity, mitochondrial membrane potential and membranes integrities indicate that this enzyme acts as a buffer to maintain the oxi-reduction and cellular homeostasis equilibrium during cryopreservation. In the scenario of fresh semen (physiological condition), SOD correlates positively with HMP. High mitochondrial membrane potential seems to favor ROS production, specially by complex III of mitochondrial electron transport chain [53]. In fact, 1–2 % of the oxygen consumed in the oxidative phosphorylation is converted into superoxide anion [54]. In the cooling condition, SOD is maintained as a metabolism buffer enzyme, correlating negatively with MMP and positively with IMIA. On the other hand, in the thawed group, when the damage is established, the correlation between SOD and IMIA inverses (positive to negative) indicating that its role as buffering is no longer enough to maintain cellular integrity. Our results highlight the contribution of the antioxidant enzymes to support sperm metabolism. However, during the cryopreservation process such enzymes are probably not sufficient to avoid oxidative cryodamages.

## Conclusion

In conclusion, cryopreservation process can damage sperm cell in different compartments such as membranes (plasma and acrosome), mitochondria and even chromatin damages, without recovery after 2 hs of incubation. During this process, the critical moment is when sperm are subjected to freezing temperatures. In addition, our study indicates that intracellular antioxidant machinery of sperm cell (SOD and GPX enzymes) is not enough to control cryodamage.
